# T74. ACADEMIC ACHIEVEMENT AND SCHIZOPHRENIA: A META-ANALYSIS

**DOI:** 10.1093/schbul/sby016.350

**Published:** 2018-04-01

**Authors:** Hannah Dickson, Alexis Cullen, Sheilagh Hodgins, James MacCabe, Kristin Laurens

**Affiliations:** 1 Institute of Psychiatry, Psychology & Neuroscience, King’s College London; 2 University of Montreal; 3 Australian Catholic University

## Abstract

**Background:**

The extent to which poor academic achievement is associated with later schizophrenia is unclear. The aim of the present study was to update our prior meta-analyses which examined academic achievement in youth aged 16 years or younger who later developed schizophrenia or schizophrenia spectrum disorders (SSD) and those who did not (Dickson et al, 2012, Psychological Medicine, 42, 743–755). We also conducted a new meta-analysis on published studies that reported on general academic achievement in youth at-risk for schizophrenia/SSD aged 16 years or younger compared to typically developing youth

**Methods:**

In addition to the five studies included in our earlier meta-analyses, a further three prospective investigations of birth or genetic high-risk cohorts were identified that reported results using objective measures of general academic achievement and of mathematics achievement for individuals who did and did not develop schizophrenia/SSD in adulthood. For our new meta-analysis we identified a total of seven studies that met the following inclusion criteria: (1) written in English; (2) objective measure of general academic achievement consisting of scores on least two core academic subjects (i.e., literacy and mathematics) at age 16 years or younger; (3) results provided for youth at high risk for developing schizophrenia/SSD in adulthood by virtue of having at least one first-degree relative with the disorder or reporting psychotic like-experiences (PLEs); and (4) sufficient data to calculate effect sizes.

**Results:**

Meta-analyses showed that by age 16 years, individuals who later developed schizophrenia/SSD presented with significantly poorer general academic achievement (d=-0.26) and mathematics achievement (d=-0.21). Findings also indicated that during adolescence, youth with a family history of schizophrenia/SSD and youth reporting PLES were characterised by significantly lower general academic achievement than healthy peers (d=-0.39; d=-0.53, respectively).

**Discussion:**

These results show that poor academic achievement precedes illness onset, and may represent an easily identifiable non-specific marker of biological, psychological and social risk processes underpinning the development of schizophrenia/SSD.

